# Effects of Repetitive Transcranial Magnetic Stimulation on Cognitive Function in Patients With Stress-Related Depression: A Randomized Double-Blind fMRI and ^1^H-MRS Study

**DOI:** 10.3389/fneur.2022.844606

**Published:** 2022-04-15

**Authors:** Yuxin Chen, Xiuzhen Li, Lubin Wang, Shushi Tian, Yuanwang Chen, Feng Wang, Kesheng Gu, Ying Wang, Guangkai Xu, Shangrong Zhang, Jie Liu, Haipeng Wang, Zongxin Jia, Liqing Li, Xiaohui Wang, Fang Xie, Xue Wang, Shida Wang, Cong Xue, Yun Zhao, Lingjia Qian

**Affiliations:** ^1^Laboratory of Stress Medicine, Beijing Institute of Basic Medical Sciences, Beijing, China; ^2^Department of Psychiatry, Hospital 984 of PLA, Beijing, China; ^3^Biochemical Laboratory, Hospital 984 of PLA, Beijing, China; ^4^Department of Imaging, Hospital 984 of PLA, Beijing, China

**Keywords:** stress, cognitive function, rTMS, fMRI, neurotransmitter

## Abstract

**Objectives:**

To reveal the effects of repetitive transcranial magnetic stimulation (rTMS) on the improvement of cognitive function in patients with stress-related depression, and to enrich the neural mechanism(s) underlying rTMS so as to improve cognitive function in patients with stress-related depression.

**Methods:**

We conducted a randomized, double-blind, placebo-controlled study of rTMS in patients with stress-related depression who were 18–40 years of age. Patients were randomly allocated to either a sham or experimental group in a 1:1 ratio. A 10-session rTMS protocol was used with 10-Hz stimulation over the left dorsolateral prefrontal cortex (DLPFC). Clinical assessments (HAMD, HAMA, DASS, MoCA), neuropsychologic (Stroop, WCST), and resting state fMRI and ^1^H-MRS assessments were executed at two time points—baseline and after the 10th rTMS session.

**Results:**

rTMS relieved the mental symptoms of patients in both groups. The MoCA score of patients in the experimental group increased; the number of correct answers increased significantly in Stroop testing, and the number of errors and omissions decreased significantly; the number of persistent errors decreased significantly; and the time used to complete the test decreased to an even greater extent in the WCST experimental group. The ReHo value in the lingual gyrus of the right hemisphere and the cuneus of the left and right hemispheres in the experimental group decreased after treatment. The DC value in the left and right hemispheric cuneus and postcentral gyrus of the left hemisphere in the experimental group diminished after treatment. The functional connections of these brain regions also changed as the Cho and NAA/Cr of the left DLPFC changed, with alterations related to the improvement in cognitive function. The level of choline (Cho) in the left DLPFC of the experimental group was significantly lower than that of the control group, and the level of N-acetylaspartate/creatine (NAA/Cr) in the left DLPFC of the control group was significantly higher than that of the experimental group. These changes were related to the overall improvement in cognitive function.

**Conclusions:**

Ten-Hz rTMS over the left DLPFC improved the cognitive function of patients with stress-related depression. The governing mechanism for this phenomenon may be via rTMS effects on multiple visual-related brain regions and their functional connections, and on the somatosensory cortex and its functional connection with visual and auditory cortex, reducing the level of Cho and stabilizing the level of NAA/Cr in the left DLPFC.

## Introduction

Depression is one of the most common mental disorders, and its estimated global prevalence is 4.4% (1), and because of its high prevalence and social cost, it is a major focus of psychiatric research. Current research suggests that depression is caused by a combination of genetic, biological, environmental, and psychological factors. However, its underlying pathophysiologic mechanisms are still not fully understood.

Stress is a state of physical and mental tension where the body responds to a variety of adverse factors in the living environment, and studies have confirmed that stress is an important biologic cause of a variety of mental disorders. When prolonged emotional stress becomes severe, it can cause depression. When a person encounters a stressful event or situation, it could trigger a depression. The feelings of depression make it very difficult to manage and deal with stress (2, 3). Intense or long-term stress may induce depression, and many patients with stress-related depression suffer from cognitive impairment.

Repetitive transcranial magnetic stimulation (rTMS) is being used increasingly in the treatment of psychiatric disorders. High frequency rTMS applied over the left dorsolateral prefrontal cortex (DLPFC) has been shown to effectively treat depression and to potentially lead to cognitive improvement as a consequence of mood amelioration. However, whether rTMS can improve the cognitive function of patients with depression remains controversial, and its underlying regulatory mechanism(s) is arcane (4–6). Some authors have demonstrated that patients with depression in an active rTMS group improved significantly on a test of cognitive flexibility, conceptual tracking, and speed performance (4, 7). However, others have uncovered no change in neuropsychologic functioning after receiving rTMS treatment for major depression (8).

In the present study, we used 10-Hz rTMS over the left DLPFC to treat patients with stress-related depression in order to explore the effects of rTMS on their cognitive function. Furthermore, we executed fMRI and ^1^H-MRS to explore the possible mechanism(s) by which rTMS improved cognitive function.

## Materials and Methods

### Study Design

Our patients first went through one week of health screening and tests—including clinical assessments, neuropsychologic testing, resting-state fMRI, and ^1^H-MRS. This was followed by two weeks of therapy that included daily treatment with active rTMS or sham stimulation (patients were required to remain on their original medication regimen throughout the study). Our protocol was a sham-controlled, randomized (1:1), double-blinded study. Patients were assigned to treatment arm using dynamic randomization (9) and stratified by sex, age, education, duration of illness, duration of hospitalization, first test score on the Hamilton depression rating scale (HAMD), Hamilton Anxiety Scale (HAMA), and Montreal Cognitive Assessment (MoCA). During treatment, one rTMS session was scheduled daily for five consecutive days, with a total of 10 sessions delivered over the two-week treatment period. After 10 sessions of therapy, all patients completed their second clinical assessment, neuropsychologic tests, resting-state fMRI, and ^1^H-MRS over one week. All of the participants were on antidepressant medications at stable doses for at least two weeks prior to, during, and one week after rTMS treatment.

### Characteristics of Participants

#### Participants

The following participants were included:

1. who were between the ages of 18 and 40 years;
2. who experienced stress prior to being diagnosed with depression;
3. who were stable after a dose of antipsychotic drugs, with a MoCA test score < 26; and
4. who were right-handed.

Patients with the following were excluded:

1. psychosis, stroke, history of seizure, head injury, or having undergone brain surgery;
2. an implanted metallic or electronic device carrier such as a cardiac pacemaker, stents, epidural or deep brain electrodes, cochlear implants, drug-infusion systems, or intracranial clips;
3. active suicidal ideation or recent suicide attempts;
4. previous substance abuse or dependence;
5. antisocial behavior or borderline personality disorder;
6. inability to complete the assessment—such as manifesting aphasia, hemiplegia, deafness, parachromatoblepsia, or hypochromatopsia;
7. current pregnancy.

**Figure 1:**
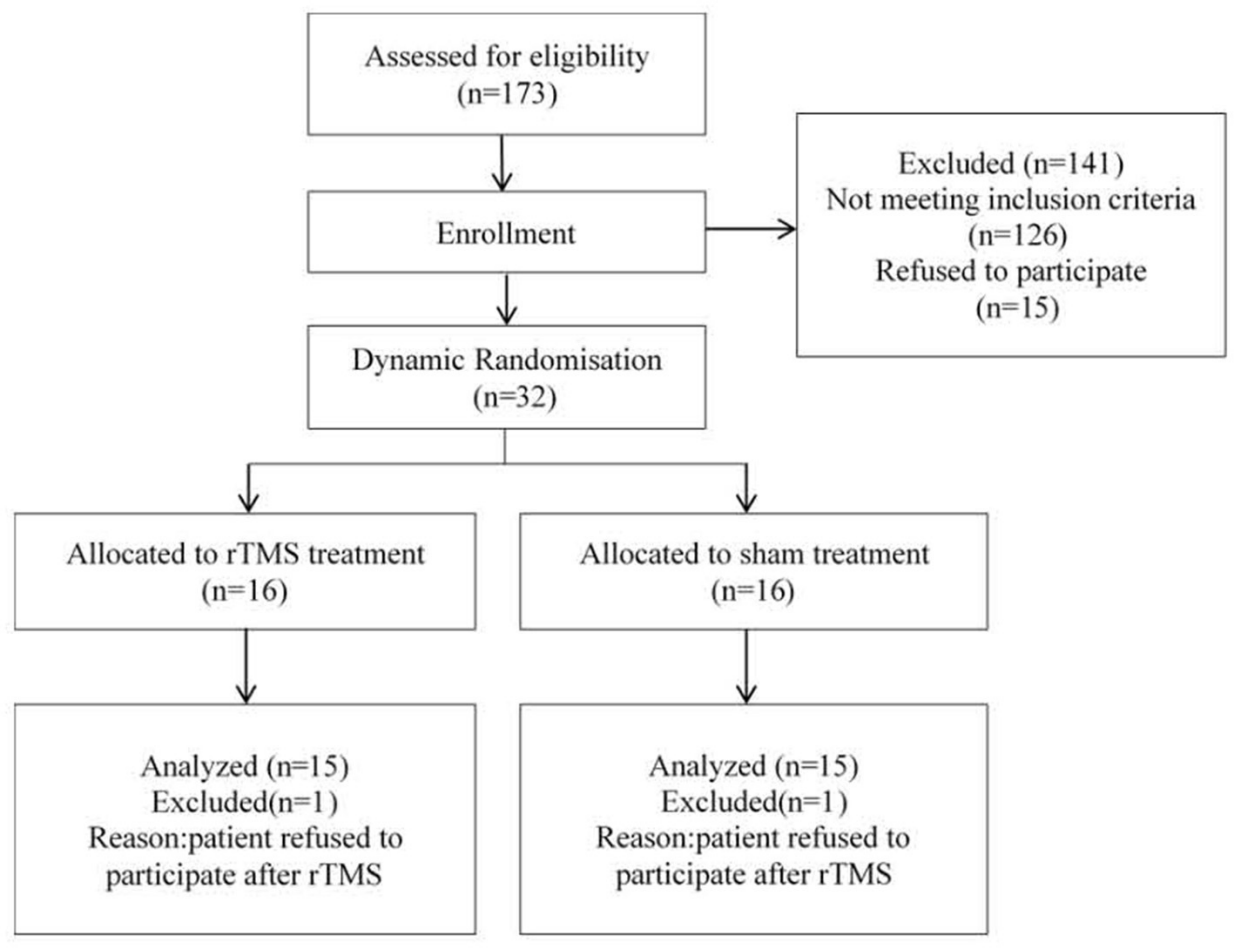
Participant flowchart.

Thirty-two right-handed patients participated in the study. All of the participants met criteria for unipolar (MDD, major depressive disorder) or bipolar (BD) illness and a current major depressive episode (MDE), as determined in a semi-structured clinical interview according to the Structured Clinical Interview for ICD-10. Two patients were withdrawn from the study after the rTMS series because they refused to participate. For an overview of the clinical trial, see Figure 1.

The study was performed in accordance with the Declaration of Helsinki and was approved by the Local Ethics Review Committee (Ethics Committee of the Hospital 984 of PLA). All of the participants provided written informed consent after the nature of the procedures had been satisfactorily explained.

### rTMS Parameters and Session Procedures

For rTMS, we used a Magstim Super Rapid Magnetic Stimulator (Magstim Company Limited, Dyfed, Wales, UK) and a high-powered, figure-8-shaped magnetic coil 7 cm in mean diameter. The rTMS intervention was then conducted over a period of two weeks (10 total sessions, one session/weekday) at 10 Hz, with 80% of the individual RMT, and in four-second trains with a 56-second inter-train interval (800 pulses per session; 8,000 total pulses per patient). Single-pulse TMS was used to measure the resting motor threshold (RMT) for the Abductor Pollicis Brevis (APB) muscle using electromyographic recording. The RMT was defined as the minimum stimulator intensity that evoked a peak–peak amplitude Motor Evoked Potential (MEP) of >50 u Vin at least five out of ten consecutive trials (10, 11). Coil position over the left DLPFC was assured in the active and sham groups by a coil-positioning method using the 10–20-EEG system. In brief, the standard 10–20 EEG electrode positions were individually measured and marked, and the position of the coil center was then located at the electrode position F3 (left posterior middle frontal gyrus, BA 46) (12, 13). For active stimulation, the coil was placed at the same point tangentially to the skull, and oriented in a posterior to anterior direction. Sham treatment was delivered in the same manner as actual TMS, but with the coil angled at 90° away from the surface of the scalp. In order to minimize the side effects to patients, rTMS risk assessment was carried out for each patient prior to treatment by a 13 items questionnaire. If the patient exhibited uncomfortable symptoms during rTMS, including headache, hearing impairment, tinnitus, etc.,we withdrew them from the study in accordance with departmental procedures.

### Neuropsychologic and Clinical Assessments

#### Clinical Assessments

To evaluate the clinical symptoms such as depression and anxiety and cognitive function, the Hamilton Rating Scale for Depression (14) and the Hamilton Anxiety Scale were conducted (15). Additional measures of symptom severity included the 21-item Depression Anxiety Stress Scale (DASS-21) (16), and all subjects completed the Montreal Cognitive Assessment (MoCA) (17). Patients were assessed at baseline (one week before rTMS treatment) and at the end of the treatment period (one week after the last session of the rTMS treatment).

#### Neuropsychologic Testing

To assess the patient's executive function, two neuropsychologic tests (the WCST and Stroop test), lasting ~30 min, were administered to each patient one week before and one week after treatment. We employed the computer-based Wisconsin Card Sorting Test (WCST), which is commonly used to assess cognitive flexibility and abstract reasoning (18, 19), and the Stroop Test was implemented to assess selective attention, set shifting, and response inhibition (20, 21).

### MRI Data Acquisition

#### Image Data Acquisition

In order to analyze the regulatory mechanism of rTMS on the spontaneous activity of brain neurons, we used an fMRI scanning procedure for functional MRI, and axial gradient-echo echo-planar imaging (EPI) to acquire images on a 3.0 Tesla Philips whole-body imaging system. Images were obtained using a standard 32-channel-head coil, where the head was stabilized with small cushions to minimize movement. During scanning, all patients were asked to keep their eyes shut but not fall asleep, remain composed, and attempt to produce no systematic cognitive or motor activity.

An EPI sequence was applied to collect resting-state functional images using the following parameters: slices = 36; slice thickness = 4 mm, slice gap = 0 mm; TE = 25 ms, TR = 2,000 ms, flip angle (FA) = 90, field of view (FOV) = 240 × 240 mm^2^, matrix = 64 × 64, and voxel size = 2 × 2 × 4 mm^3^. We collected 180 time points from each subject. A set of high-resolution T1-weighted structural images was collected by applying a three-dimensional fast spoiled gradient-echo (3DSPGR) sequence with the following parameters: slices = 124; slice thickness = 1.6 mm, slice ga*p* = 0 mm; TE = 2.8 ms, TR = 450 ms, FA = 15, FOV = 240 × 240 mm^2^, matrix = 256 × 256 and isotropic voxel size = 1.6 × 1.6 × 1.6 mm^3^.

#### Image Data Preprocessing

MRI data were preprocessed using the Data Processing Assistant for Resting-State fMRI Advanced Edition (DPARSF) (22), which is based on MATLAB (2013b) and SPM8. Because of the time required for magnetization equilibrium and participant adjustment to a new and noisy environment, the first 10 volumes of each functional image were deleted. The remaining 170 functional images were slice-time corrected to reduce the differences in images from different times and realigned for head motion. Subjects whose maximal head motion was beyond 2.0 mm in any direction or whose maximal head rotation was beyond 2.0° in any angular dimension were excluded. The high-resolution T1-weighted structural images were then co-registered with the functional images. Next, the entire brain was segmented into gray matter (GM), white matter (WM), and cerebrospinal fluid (CSF). Subsequently, we implemented a regression of nuisance covariates, including Friston 24-parameter correction and head-motion scrubbing with thresholds of ~0.5 mm; noisy signals from the CSF and WM were also regressed. Retaining or removing global brain signals is controversial; we herein chose to retain the global signals. All the data were then normalized into the standard Montreal Neurological Institute (MNI) template, and each voxel size was resampled at 3 × 3 × 3 mm and filtered at the 0.01–0.08-Hz band. Finally, we set a Gaussian kernel of 6-mm full-width at half-maximum (FWHM) to conduct spatial smoothing. In addition, detrending was used to remove the linear trends. The whole-brain ALFF was calculated for each subject using the preprocessed images with temporal band-pass filtering (0.01 < f < 0.08 Hz), which reduced low-frequency drift and high-frequency respiratory and cardiac noise. The subject-level voxel-wise ALFF map was converted into a z-score map by subtracting the mean ALFF of the whole brain and dividing by the standard deviation. The fractional low-frequency amplitude (fALFF) was obtained by dividing the ALFF signal-power spectrum by the signal-power spectrum of the entire frequency band. The fALFF value was then stronger relative to noise, with higher sensitivity and specificity (23). Regional homogeneity (ReHo) (24), degree centrality (DC) (25), and functional connectivity (FC) were also used to investigate the mechanism(s) driving rTMS effects in patients with depression.

### ^1^H-MRS Data Acquisition and Quantification Protocol

Brain neurotransmitter abnormality is an important cause of mental diseases. ^1^H-MRS is a non-invasive technique to determine the chemical composition of specific tissue areas in vivo. Magnetic resonance spectrum imaging (MRSI) data were examined using a 3.0 Tesla Philips whole-body imaging system for all patients. The MRI protocol included T1-weighted, 3D spoiled-gradient echo acquisitions in transaxial and coronal orientations, and T2- and a fluid-attenuated inversion recovery sequence in sagittal orientation. A water-suppressed, chemical shift-imaging spine echo sequence was executed using multiple voxel proton MRS. Parameters were described as follows: echo time = 135 ms, repetition time = 2,000 ms, NSA = 128, and voxel size = 1.5 × 1.5 × 1.5 cm^3^. Acquisition was repeated three times, with the voxel sitting in region to reduce the errors resulting from partial volume effects and to improve the signal-to-noise ratio. MRSI scans that covered the primary ROIs (left posterior middle frontal gyrus, in the BA 46) were referred to readily identifiable locations on each subject's matching high-resolution MR images (Figure 1). Head motion was minimized by comfortably securing subjects' heads with padding within the quadrature head coil.

**Figure 2:**
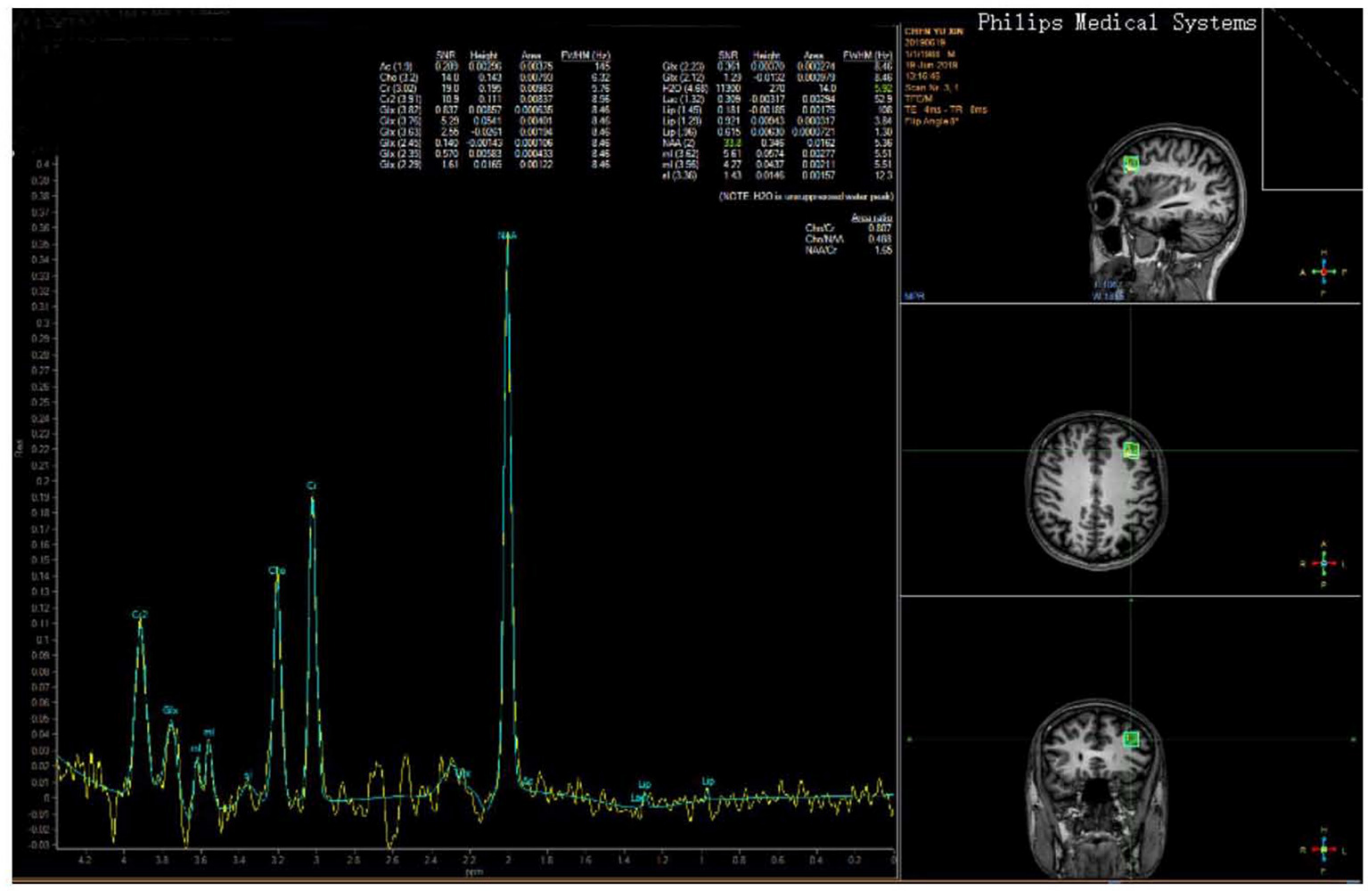
A single voxel was placed in the left DLPFC (in BA 46, shown at right). A typical spectrum from this voxel is shown on the left and demonstrates metabolite peaks for N-acetyl-aspartate (NAA), choline (Cho), and creatine (Cr), which have been implicated in the pathophysiology of depression.

A representative spectrum of a patient from the left posterior middle frontal gyrus MRSI voxel is shown in Figure 1. Concentrations in proton MRS studies are expressed relative to creatine, which acts as an internal reference standard in the voxel. We evaluated each spectrum for the peak area of NAA, Cho, and Cr, and also calculated the ratios of NAA/Cr and Cho/Cr. The post-processing and quantification were performed automatically with the Philips brain imaging software package. Spectral post-processing comprised line broadening, reducing the residual water resonance, linear baseline correction, and peak integration (Figure 2).

### Data Analysis

#### Neuropsychologic and Clinical Assessments, and Metabolite Data Analysis

We employed SPSS v. 24.0 statistical software to analyze the data. The balance of the two groups of patients was tested as follows: for continuous variables such as scale scores, we used an independent-sample *t* test; for naming variables such as medication and sex, we used a Chi-squared test. Treatment effects data were entered into a 2 (time of measurement, pre-treatment vs. post-treatment) × 2 (stimulation condition, active vs. sham) factorial design, with time of measurement as the within-subjects factor. Thus, for each test variable, we performed an ANOVA for repeated measures with additional post-hoc analyses using paired, two-tailed *t* tests in the case of significant results indicated by interactions in the ANOVA, with all *p*-values corrected for multiple comparisons. The scale scores and statistical significance were assigned at *P* < 0.05. Pearson's correlation analysis was applied to investigate relationships among clinical ratings (HAMD, HAMA, DASS-21, MoCA), cognitive performance (Stroop, WCST), and metabolites.

#### Neuroimaging Analyses

Resting fMRI index reflects the level of spontaneous activity of brain neurons in resting state, which is more suitable for the study of patients with stress disorder without clear organic lesions. The change of synchronization or difference of brain activity (network function connection characteristics, etc.) may be the main reason for the abnormality of cognitive function and emotional state. Increases in BOLD activity correlated with increases in neuronal activity, making it possible to estimate the amount and anatomic location of brain activity that occurred during a particular pathological state. Regional Homogeneity (ReHo) reaction the activity of voxel is consistent with that of its surrounding voxel neurons. Degree centrality (DC) indicates the strength of the connection between this region and other voxels in the whole brain. Fractional Amplitude of Low Frequency Fluctuations (fALFF) can reflect the strength of brain neuron activity from the perspective of energy. We analyzed the ReHo, DC, ALFF, and FC maps with DPARSF. Age, sex, and educational level were used as covariates in two-sample *t* test calculations. We used the Gaussian random field (GRF) to correct for multiple comparisons. For all of the aforementioned analyses, we set a voxel-level threshold of *p* = 0.001 and a cluster-level threshold of *p* = 0.05, and GRF correction was applied to multiple comparisons in the whole brain. In addition, a GM group mask was employed in ReHo, DC, fALFF, and FC calculations.

A seed-based, functional-connection analysis method selects significant differences in areas of ReHo, DC, and fALFF as seed points. The peak points of the ReHo, DC, and fALFF analyses were then selected as the coordinates of those regions of interest (ROIs), and the radius was set at 6 mm. We calculated seed-based FC maps between the time-courses of seed regions and the time-series for all voxels in the global brain with Pearson's correlation analyses. Finally, Fisher's *r*-to-z transformation was applied to all maps prior to statistical analysis. Pearson's correlation coefficients were calculated to assess relationships among clinical measures, neuropsychological results, ReHo, DC, ALFF, and FC maps in patients. Calculations were performed using SPSS software after eliminating the influences of age, sex, and education, and statistical significance was set to *p* < 0.05.

## Results

Thirty-two subjects completed the first MRI and 30 completed both scans. Subjects tolerated the rTMS treatments well, and there were no serious adverse events.

### Demographic and Clinical Characteristics

There was no significant difference in demographic information, scale baseline, antipsychotic drug use, risk factors, or biochemical indices in blood between the two groups (Table 1).

**Table 1:** Clinical and socio-demographic information of study participants.

	**Experimental rTMS (*n* = 15)**	**Sham rTMS (*n* = 15)**	**Statistic**	***p***
**Demographic characteristic**				
Sex (male/female)	15/0	14/1	1.034	0.309
Age (years)	26.47 (6.96)	28.80 (4.94)	−1.059	0.299
Education (years)	12.07 (1.79)	13.67 (2.74)	−1.891	0.069
Duration of illness (months)	22.93 (22.27)	28.33 (22.75)	−0.657	0.517
Duration of hospitalization	2.53 (3.79)	2.77 (3.02)	−0.107	0.916
**Baseline psychopathology**				
HAMD	12.07 (9.06)	13.20 (9.37)	−0.337	0.739
HAMA	7.80 (7.15)	11.00 (8.98)	−1.080	0.289
DASS	66.14 (28.72)	64.80 (29.84)	0.125	0.902
MOCA	20.93 (3.51)	21.93 (2.87)	−0.854	0.400
**Concomitant medication (taken/not taken)**				
Sodium valproate	3/12	4/11	0.186	0.666
Olanzapine	3/12	2/13	0.240	0.624
Sertraline	3/12	0/15	3.333	0.068
Tandospirone	5/10	6/9	0.144	0.705
Dutoxetine	7/8	6/9	0.136	0.713
Mitrazapine	9/6	7/8	0.536	0.464
Oxazepam	3/12	1/14	1.154	0.283
Lithium carbonate	0/15	2/13	2.143	0.143
Escitalopram oxalate	2/13	1/14	0.370	0.543
Sulpiride	0/15	1/14	1.034	0.309
Trazodone	2/13	2/13	0.000	1.000
Quetiapine fumarate	0/15	3/12	3.333	0.068
Lorazepam	1/14	2/13	0.370	0.543
Zilacetone hydrochloride	0/15	1/14	1.034	0.309
Venlafaxine	1/14	0/15	1.034	0.309
**Risk factors**				
Hcy (μmol/L)	13.84 (6.84)	13.96 (6.31)	−0.050	0.961
Family history of mental illness	2/13	1/14	0.370	0.543

*Student's t test for continuous data and χ^2^ for categorical data*.

*Values in brackets reflect standard deviation of the mean*.

### Clinical Characteristics and Neuropsychologic Results

In this study, we uncovered a significant interaction between the total score for the MoCA and time (*p* = 0.006). After rTMS, the total score for the MoCA in the experimental group increased significantly, but there was no significant change in the control group. The scores for short-term memory and orientation to time and place (MoCA sub-factors) in the experimental group were significantly improved after rTMS (*p* < 0.05), while the scores for other sub-factors experienced no significant change. The scores for all sub-factors also underwent no significant change in the control group.

The interaction between group and time for all indices in the Stroop test was not significant. The correct numbers in the Stroop test in the experimental group increased significantly, while the numbers of errors and omissions decreased significantly (*p* < 0.05) after rTMS, and other indices did not change.

The interaction between group and time in the numbers of perseverative errors and time to complete the WCST was significant (*p* = 0.048, *p* = 0.050), and the number of perseverative errors in the experimental group decreased significantly after rTMS with no significant change in the control group. The time to complete the test by patients in both groups diminished significantly (*p* < 0.01), although the experimental group declined to a greater degree (*p* = 0.001). The total response numbers for patients in the experimental group and the number of responses needed to complete the first classification decreased significantly after rTMS (*p* < 0.05), while there was no significant change in the control group.

The interaction between group and duration of depression, anxiety, and stress subscale of the DASS, HAMD, and HAMA was not significant, but the main effect of time was significant. After treatment, the scores for depression, anxiety, and the stress subscale of the DASS, HAMD, and HAMA in the experimental group decreased significantly, while in the control group, except for depression subscale of the DASS, the scores for the other scales also decreased significantly. We suggest that the symptoms of depression and anxiety in both groups were relieved after treatment (Table 2).

**Table 2:** Clinical characteristics and neuropsychologic assessments of the participants.

	**Treatment group (*****n*** **= 15)**		**Control group (*****n*** **= 15)**		***p* value, group**	***p* value, time**	***p* value interaction (time × group)**
	**Pre-rTMS**	**Post-rTM**	**Pre-rTMS**	**Post-rTMS**			
	**Mean (SD)**	**Mean (SD)**	**Mean (SD)**	**Mean (SD)**			
**Anxiety and depression**							
HAMD score	12.07 (9.06)	5.73 (5.04)^a^	13.20 (9.37)	5.20 (3.59)^a^	0.894	0.000	0.556
HAMA score	7.80 (7.15)	3.07 (3.56)^a^	11.00 (8.98)	4.13 (3.02)^a^	0.264	0.000	0.41
DASS-depression score	22.80 (10.58)	15.60 (10.80)^a^	22.26 (10.00)	15.34 (11.86)	0.908	0.001	0.946
DASS-anxiety score	18.00 (10.02)	10.94 (8.34)^a^	19.20 (11.36)	12.80 (10.34)^a^	0.640	0.001	0.855
DASS-stress score	25.34 (10.66)	16.40 (11.88)^a^	23.34 (10.90)	13.60 (12.72)^a^	0.526	0.000	0.841
**MoCA**							
Total points	20.93 (3.51)	24.67 (2.99)^a^	21.93 (2.87)	22.77 (2.76)	0.614	0.000	0.006
Visuospatial abilities	3.26 (0.59)	3.40 (0.63)	2.77 (0.89)	2.53 (0.83)	0.006	1.000	0.263
Language	3.60 (0.99)	3.93 (1.09)	3.53 (0.99)	4.00 (0.93)	1.000	0.017	0.677
Executive functions	2.07 (1.39)	2.80 (0.86)	2.40 (1.12)	2.70 (0.98)	0.845	0.045	0.239
Attention	5.47 (0.74)	5.67 (0.49)	5.33 (1.11)	4.93 (1.09)	0.135	0.559	0.087
Short-term memory	1.67 (1.35)	3.27 (1.49)^a^	2.53 (1.36)	3.07 (1.33)	0.446	0.000	0.051
Orientation to time and place	4.87 (1.06)	5.60 (0.83)^a^	5.47 (0.74)	5.47 (0.64)	0.341	0.058	0.058
**Stroop**							
Accuracy of Stroop task (%)	56.97 (21.5)	76.58 (15.68)	62.87 (24.85)	72.84 (19.54)	0.872	0.000	0.191
Number correct	68.93 (26.02)	92.67 (18.98)^a^	76.07 (30.07)	88.13 (23.64)	0.872	0.000	0.191
Number of errors	20.6 (11.47)	10.40 (6.75)^a^	18.53 (13.69)	14.53 (10.53)	0.763	0.000	0.147
Number of omissions	31.13 (15.31)	17.73 (12.53)^a^	26.20 (18.69)	18.07 (13.47)	0.642	0.000	0.321
RT stroop task (ms)	994.9 (114.2)	1061.6 (85.8)	985.4 (101.9)	1035.9 (88.2)	0.539	0.012	0.716
**WCST**							
Trials administered	115 (16.0)	102 (22.7)^a^	121 (10.9)	112 (17.4)	0.142	0.004	0.479
Perseverative errors	6.60 (2.82)	2.20 (3.28)^a^	4.80 (3.49)	3.60 (3.42)	0.827	0.001	0.048
Percent perseverative error (%)	5.68 (2.21)	2.02 (2.95)^a^	4.14 (3.18)	3.13 (2.91)	0.782	0.002	0.062
Trials to complete first category	27.3 (19.4)	14.3 (7.32)^a^	25.2 (18.1)	19.4 (10.8)	0.717	0.015	0.328
Time (s)	753.9 (372.1)	388.3 (166.7)^a^	613.9 (190.0)	449.9 (213.5)^a^	0.612	0.000	0.05

a *P < 0.05, post-rTMS compared with pre-rTMS in the same groups*.

### Cognitive Function and Mental-Symptom Outcomes

In this study, we discovered that the interaction between the total score for the MoCA and time was significant (*p* = 0.006). After rTMS, the total score for the MoCA and the scores of short-term memory and orientation to time and place (MoCA sub-factors) in the experimental group increased significantly (*p* < 0.05), while there was no significant change in the control group.

The interaction between group and time for all indices in the Stroop test was not significant. The correct numbers in the Stroop test in the experimental group increased significantly, while the numbers of errors and omissions decreased significantly (*p* < 0.05) after rTMS, and other indices did not change.

The interaction between group and time in the number of perseverative errors and time to complete the WCST was significant (*p* = 0.048, *p* = 0.050), and the number of perseverative errors in the experimental group decreased significantly after rTMS. However, there was no significant change in the control group. Moreover, the time to complete the test by patients in both groups decreased significantly (*p* < 0.01), although the improvement was more pronounced in the experimental group (*p* = 0.001). The total response numbers by patients in the experimental group and the number of responses needed to complete the first classification decreased significantly after rTMS (*p* < 0.05), while there was no significant change in the control group.

The interaction between group and the duration of depression, anxiety, and the stress subscale of the DASS, HAMD, or HAMA was not significant; however, the main effect of time was significant. After treatment, the scores for depression, anxiety, and the stress subscale of the DASS, HAMD, and HAMA in the experimental group decreased significantly, and in the control group—except for the depression subscale of DASS—the scores of the other scales were also reduced significantly. We posit that the symptoms of depression and anxiety in the two groups were relieved after treatment (Table 2).

### ReHo, DC, and fALFF Analyses

fMRI is a useful technology that measures changes in regional CNS blood oxygenation in parallel with regional metabolic activity. Elevations in blood-oxygenation-level-dependent (BOLD) activity correlated with increases in neuronal activity, making it possible to estimate the amount and anatomic location of brain activity that occurred during a particular pathologic state.

**Figure 3:**
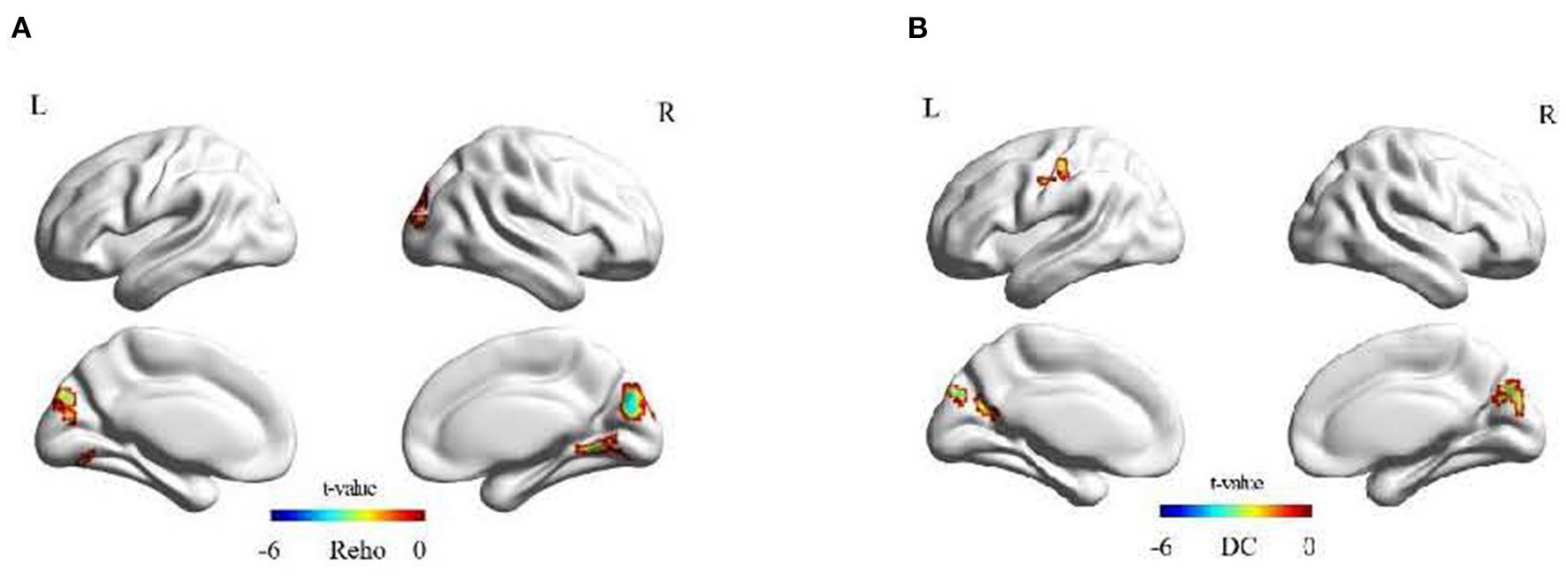
Significant changes in the experimental group after rTMS. **(A)** Significant changes in ReHo values in the experimental group after rTMS. **(B)** Significant changes in DC values in the experimental group after rTMS. The color bar represents the *t* value of the paired t test.

We noted attenuated ReHo values under a resting state in the lingual gyrus of the right hemisphere and the cuneus of the left and right hemispheres in the experimental group after rTMS, there was no significant change in the ReHo value in the control group. The DC values that showed diminutions in the experimental group after rTMS were the postcentral gyrus of the left hemisphere, and the cuneus of the left and right hemispheres, but there was no significant change in the DC of the control group (Figure 3), nor in fALFF. We observed no significant difference using an independent-sample *t* test between the two groups at the same time (pre- or post-rTMS). Neither the main effects of group and time nor the interaction group × time was significant (Table 3, Figure 3).

**Table 3:** ReHo and DC alterations after rTMS treatment in the experimental group.

**Brain region**	**BA**	**Hem**	**MNI peak-point coordinates**			**Voxels**	***t* value**
			**x**	**y**	**z**		
**ReHo**							
Lingual gyrus	18	R	9	−60	−3	118	−6.7456
Cuneus	18, 19	L, R	18	−84	33	202	−5.0873
**DC**							
Postcentral gyrus	4	L	48	−21	39	69	−6.0176
Cuneus	18, 19	L, R	15	−81	27	122	−5.5859

*Gaussian random field (GRF) corrected; voxel p value < 0.001; cluster p value < 0.05; two-tailed*.

### FC Analysis

**Figure 4:**
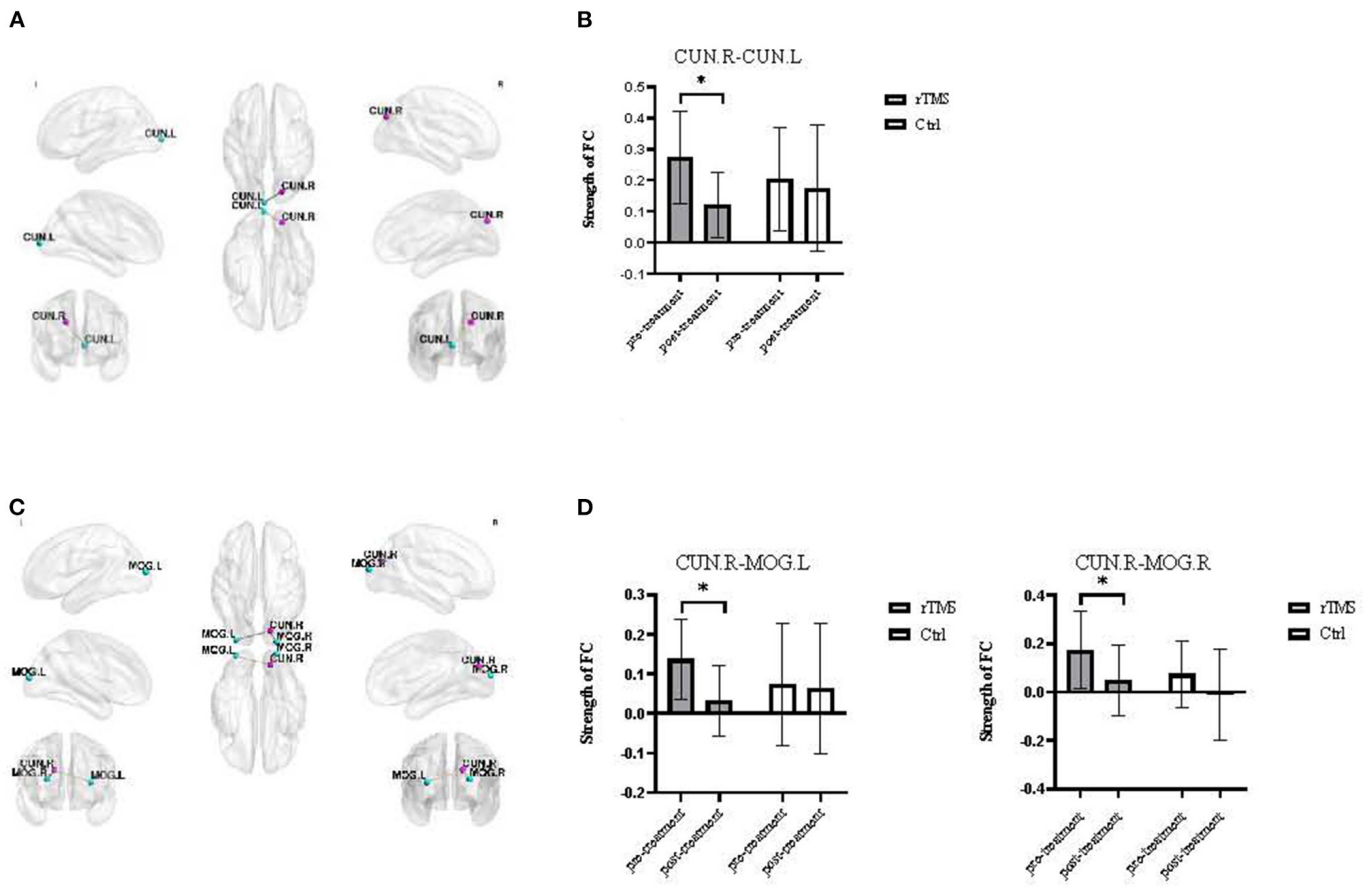
Significantly altered FC after treatment in the rTMS group (voxel *p* < 0.001, cluster *p* < 0.05, two-tailed, GRF-corrected). **(A)** The results of functional connectivity analyses with the seed region located in the CUN.R (18 −84 33). **(B)** The panel displays the FC strength values from CUN.R to CUN.L. **(C)** The results of the functional connectivity analyses with the seed region located in the CUN.R (15 −81 27). **(D)** The panel displays the FC strength values from CUN.R to MOG.L and MOG.R. CUN, cuneus; MOG, middle occipital gyrus. *P < 0.05, Post-rTMS compared with pre-rTMS in the same groups.

**Figure 5:**
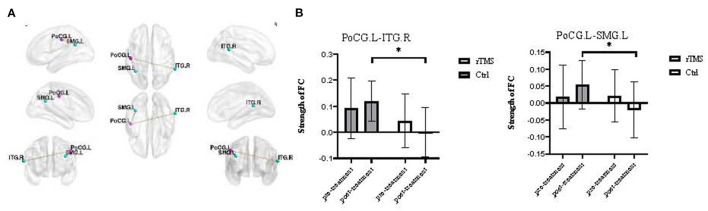
Significantly different FC after treatment between the two groups (voxel *p* < 0.01, cluster *p* < 0.05, two-tailed, GRF-corrected). **(A)** The results of the functional connectivity analyses with the seed region located in the PoCG.L (−48 −21 39). **(B)** The panel displays the FC strength values from PoCG to ITG.R and SMG.L. PoCG, postcentral gyrus; ITG, inferior temporal gyrus; SMG, supramarginal gyrus. *P < 0.05, Treatment group compared with control group at the same timepoint.

Four brain regions with significant differences in ReHo and DC values were used as seed points to analyze the functional connection with other voxels of the whole brain. We found that in the experimental group, the functional connection strength of the right cuneus and left cuneus, and the right cuneus and left and right middle occipital gyrus changed significantly after rTMS. After rTMS, there was also a significant difference in the functional connection strength of the left postcentral gyrus to the inferior temporal gyrus and supramarginal gyrus between the experimental group and the control group. Using the lingual gyrus as the seed point, we did not find any significant change in functional connectivity with other voxels (Table 4; Figures 4, 5).

**Table 4:** Brain regions showing significantly altered FC.

**Seed point**	**Peak area**	**BA**	**MNI peak-point coordinates**			**Voxels**	**t Value**
			**x**	**y**	**z**		
**Significantly altered FC after treatment in rTMS group**							
Cuneus (18 −84 33)							
	Cuneus	18L	−6	−99	3	318	5.4351
Cuneus (15 −81 27)							
	Middle occipital gyrus	18, 19L	−30	−93	12	176	5.7132
	Middle occipital gyrus	18, 19R	24	−96	15	177	4.6835
**Significantly different FC after treatment between the two groups**							
Postcentral gyrus (−48 −21 39)							
	Inferior temporal gyrus	20, 37R	63	−48	15	155	5.1516
	Supramarginal gyrus	7, 39L	−36	−54	27	117	5.2488

*Gaussian random field (GRF) corrected; voxel p value < 0.001; cluster p value < 0.05; two tailed*.

### Correlations Among Clinical Data, Neuropsychologic Data and ReHo, DC, FC Values

**Figure 6:**
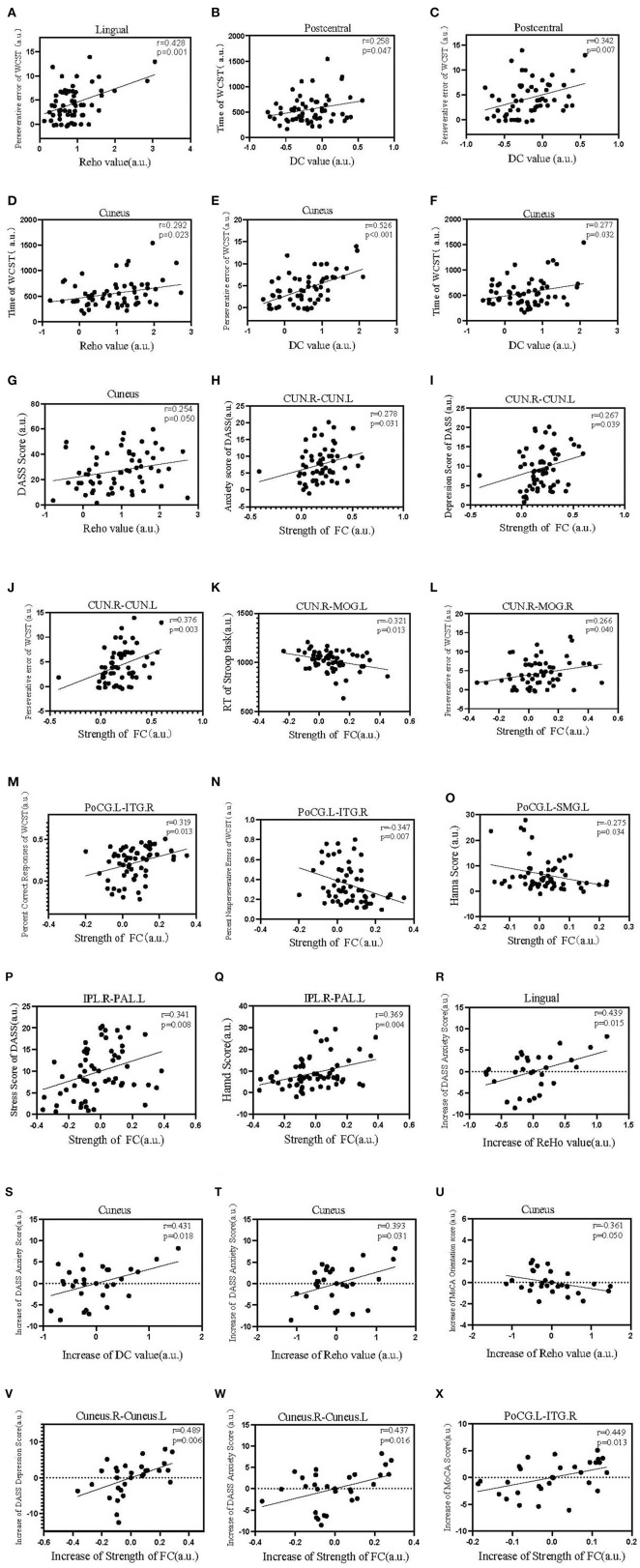
Scatter diagrams showing significant correlations among the clinical data, neurophysiologic assessments, ReHo, DC, and FC strength for all patients (pretherapy and posttherapy). **(A)** The ReHo value of the lingual region was positively correlated with the perseverative error of the WCST. **(B,C)** The DC value of the postcentral gyrus was positively correlated with the time taken for the WCST and perseverative error of the WCST. **(D)** The ReHo value of the cuneus was positively correlated with the time taken for the WCST. **(E,F)** The DC value of the cuneus was positively correlated with perseverative error of the WCST and time of the WCST. **(G)** The ReHo value of the cuneus was positively correlated with DASS score. **(H–J)** FC strength between the CUN.R and CUN.L was positively correlated with anxiety score on the DASS, depression score on the DASS, and perseverative error on the WCST. **(K)** FC strength between the CUN.R and MOG.L was negatively correlated with reaction time (RT) on the Stroop task. **(L)** FC strength between the CUN.R and MOG.R was positively correlated with perseverative error of WCST. **(M)** FC strength between the PoCG.L and ITG.R was positively correlated with percent correct responses on the WCST. **(N)** FC strength between the PoCG.L and ITG.R was negatively correlated with percent non-perseverative errors. **(O)** FC strength between the PoCG.L and SMG.L was negatively correlated with the HAMA score. **(P,Q)** FC strength between the IPL.R and PAL.L was positively correlated with stress score of the DASS and HAMA scores. **(R)** Increase in ReHo value in lingual was positively correlated with an increase in the anxiety score in the DASS. **(S)** Increase in the DC value in cuneus was positively correlated with an increase in anxiety score in the DASS. **(T,U)** Increase in the Reho value in cuneus was positively correlated with an increase of DASS Anxiety Score and negatively correlated with increase in MoCA orientation score. **(V)** Increase in FC strength between Cuneus.R and Cuneus.L was positively correlated with an increase in depression score (DASS). **(W)** Increase in FC strength between Cuneus.R and Cuneus.L was positively correlated with an increase in anxiety score (DASS). **(X)** Increase in FC strength between PoCG.L and ITG.R was positively correlated with an increase in the MoCA score.

After controlling for age, sex, and educational level, we performed a partial correlation analysis of the two group. We executed a correlation analysis of the two group of patients between ReHo, DC, and FC values and the scores on cognitive tests and psychiatric symptom scales. The ReHo value of the lingual gyrus was positively correlated with perseverative error on the WCST (*r* = 0.428, *p* = 0.001). The DC value of the postcentral gyrus was positively correlated with the time taken for the WCST and perseverative error of the WCST(*r* = 0.258, *p* = 0.047; *r* = 0.342, *p* = 0.07). The ReHo value of the cuneus was positively correlated with the time taken for the WCST(*r* = 0.292, *p* = 0.023), the DC value of the cuneus was positively correlated with perseverative error of the WCST and time of the WCST (*r* = 0.526, *p* < 0.001; *r* = 0.277, *p* = 0.032), and the ReHo value of the cuneus was positively correlated with DASS score (*r* = 0.254, *p* = 0.05). The FC strength between the CUN.R and CUN.L was positively correlated with anxiety score on the DASS, depression score on the DASS, and perseverative error of the WCST (*r* = 0.278, *p* = 0.031; *r* = 0.267, *p* = 0.039; *r* = 0.376, *p* = 0.003). FC strength between the CUN.R and MOG.L was negatively correlated with RT of the Stroop task (*r* = −0.321, *p* = 0.013), FC strength between the CUN.R and MOG.R was positively correlated with perseverative error of WCST (*r* = 0.266, *p* = 0.040). FC strength between the PoCG.L and ITG.R was positively correlated with percent correct responses to the WCST (*r* = 0.319, *p* = 0.013), and it was negatively correlated with percent non-perseverative errors (*r* = −0.347, *p* = 0.007). FC strength between the PoCG.L and SMG.L was negatively correlated with the HAMA score (*r* = −0.275, *p* = 0.034), and FC strength between the IPL.R and PAL.L was positively correlated with stress score on the DASS and HAMD (*r* = 0.341, *p* = 0.008; *r* = 0.369, *p* = 0.004). An increase in the ReHo value in the lingual gyrus was positively correlated with an increase in the anxiety score in the DASS (*r* = 0.439, *p* = 0.015); an increase in the DC value in the cuneus was positively correlated with an increase in the anxiety score in the DASS (*r* = 0.431, *p* = 0.018); an increase in the ReHo value in cuneus was positively correlated with an increase of DASS Anxiety Score (*r* = 0.393, *p* = 0.031) and negatively correlated with increase in MoCA orientation score (*r* = −0.361, *p* = 0.050). An increase in FC strength between Cuneus.R and Cuneus.L was positively correlated with an increase in the depression score (DASS) (*r* = 0.489, *p* = 0.006); an increase in FC strength between Cuneus.R and Cuneus.L was positively correlated with an increase in the anxiety score (DASS) (*r* = 437, *p* = 0.016); and an increase in FC strength between PoCG.L and ITG.R was positively correlated with an increase in the MoCA score (*r* = 0.449, *p* = 0.013) (these correlation analyses are illustrated in Figure 6).

### Metabolic Outcomes

The interaction of the three brain metabolites and their ratio between groups and time was not significant. Before rTMS, there was no significant difference in the level of choline (Cho) in the left dorsolateral prefrontal cortex between the two groups (*p* = 0.394), while after rTMS, the level of Cho in the left DLPFC in the experimental group was significantly lower than that in the control group (*p* = 0.011). Before rTMS, there was a significant difference in the ratio of N-acetylaspartate to creatine (NAA/Cr) in the left DLPFC between the two groups (*p* < 0.024), and after rTMS, the level of NAA/Cr in the left DLPFC in the experimental group was significantly higher than that in the control group (*p* < 0.001) (Table 5).

**Table 5:** Comparisons of neurotransmitters in the left DLPFC by MRS between pre- and post-rTMS treatment of the two groups.

	**Treatment group (*****n*** **= 15)**		**Control group (*****n*** **= 15)**		***p* value, group**	***p* value, time**	***p* value interaction (time × group)**
	**Pre-rTMS**	**Post-rTM**	**Pre-rTMS**	**Post-rTMS**			
	**Mean (SD)**	**Mean (SD)**	**Mean (SD)**	**Mean (SD)**			
NAA	0.0192 (0.0044)	0.0173 (0.0033)	0.0186 (0.0041)	0.0189 (0.0037)	0.632	0.433	0.270
NAA/Cr	1.7567 (0.2042)	1.8125 (0.1940)	1.6007 (0.1509)^b^	1.5449 (0.1066)^b^	0.001	1.000	0.067
Cho	0.0084 (0.0016)	0.0074 (0.0019)	0.0089 (0.0015)	0.0092 (0.0015)^b^	0.013	0.427	0.168
Cho/Cr	0.7805 (0.1153)	0.7881 (0.1804)	0.7720 (0.090)	0.7654 (0.1051)	0.702	0.982	0.760
Cr	0.0110 (0.0228)	0.0096 (0.0021)	0.0117 (0.0024)	0.0123 (0.0024)^b^	0.006	0.500	0.111

b *P < 0.05, Treatment group compared with control group at the same timepoints*.

**Figure 7:**
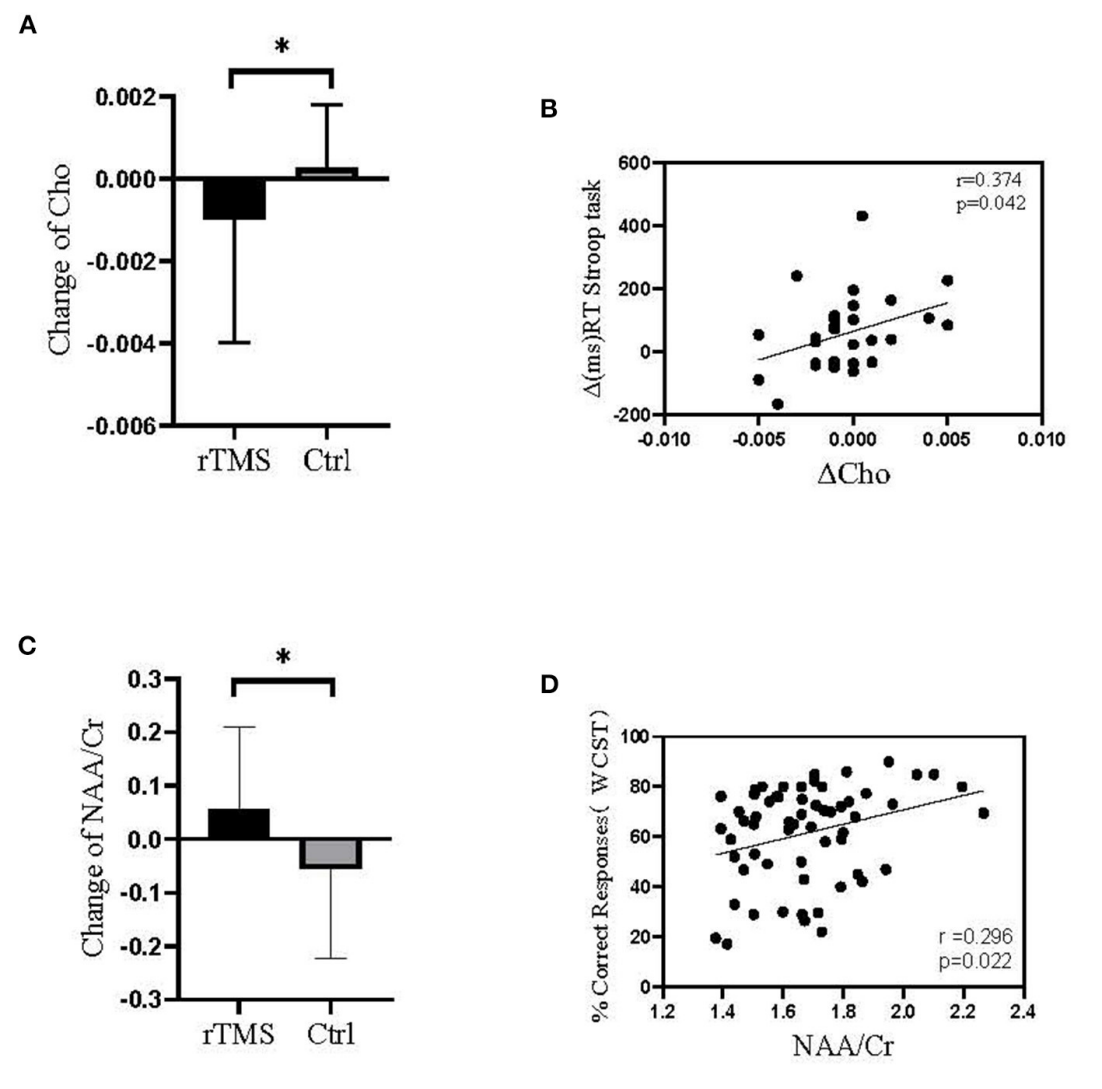
The level of Cho and NAA/Cr in the left DLPFC before and after treatment and its relationship with cognitive testing. **(A)** The change in Cho in the left DLPFC in the experimental group was significantly lower than that in the control group. **(B)** The change in Cho in the left DLPFC was positively correlated with change in the RT on the Stroop task. **(C)** The change in NAA/Cr in the left DLPFC in the experimental group was significantly higher than that in the control group. **(D)** The NAA/Cr value in the left DLPFC was positively correlated with correct response rate on the WCST. *P < 0.05, Treatment group compared with control group.

We performed a correlation analysis of the two groups of patients between the brain metabolic index and the scores on cognitive tests and psychiatric symptom scales (Figure 7). The change in Cho in the left DLPFC in the experimental group was significantly lower than that in the control group (*p* = 0.168), and positively correlated with changes in the RT Stroop task (*r* = 0.374, *p* = 0.042). The change in NAA/Cr in the left DLPFC in the experimental group was significantly higher than that in the control group (*p* = 0.067), while the ratio was positively correlated with the correct responses rate on the WCST (*r* = 0.296, *p* = 0.022).

## Discussion

### Effects of rTMS on Cognitive Function in Patients With Stress-Related Depression

Executive function reflects a series of cognitive processes that are necessary for cognitive control of behavior. The performance of patients with depression on the WCST, Stroop color word test, and other neuropsychologic tests has been shown to be worse than those in control groups (26–28).

The MoCA scores of the patients selected for this study ranged from 15 to 25, which indicates mild cognitive impairment. After rTMS, the MoCA scores of patients in the experimental group increased significantly, and the scores for short-term memory and orientation to time and place (MoCA subfactors) in the experimental group were significantly improved after rTMS. We found that after 20-Hz dTMS was focused on the DLPFC of patients with depression, there was significant improvement in the scores on spatial working memory tests in Cambridge automated neuropsychological tests (29).

The Stroop effect color word test, as a representative test of response-inhibition ability (30–32), has been widely used in patients with different diseases and of different ages (33–36). In our study, we uncovered a significant increase in the number of correct answers, while the number of errors and omissions decreased significantly in the experimental group, but there was no significant change in the control group. This illustrates the response-inhibitory capability of the patients as having been improved after rTMS. This conclusion is the same as that drawn in a previous study in which rTMS to the DLPFC in patients was used in treatment-resistant depression (TRD) (37).

In this study, we found that the number of persistent errors on the WCST in the experimental group decreased significantly after treatment, while there was no significant change in the control group, and although the test duration in the two groups decreased significantly, the experimental group decreased to an even greater extent. The number of persistent errors reflects problems such as concept formation and plasticity (29). Some researchers also found that high-frequency (at 15 Hz) rTMS stimulation over the left DLPFC in patients with treatment-resistant depression changed the performance on the WCST (38). Other investigators used intermittent theta burst stimulation (iTBS) to treat patients with treatment-resistant depression, and demonstrated that after iTBS over the left DLPFC, the correct-answer rate on the WCST and conceptual response rate increased, while the error rate declined. This indicates that iTBS exerted a positive effect on the executive function of patients with depression (39). iTBS and high-frequency rTMS stimulation can thus actively stimulate brain areas. Both our research and that of previous studies showed that the activation of the left DLPFC led to improvements in cognitive function.

### Effects of rTMS on fMRI in Patients With Stress-Related Depression

The lingual gyrus is primarily responsible for letter processing, logical analysis, and visual memory processing (40). Our study revealed that the ReHo value in the right lingual gyrus of the experimental group decreased after rTMS and was positively correlated with perseverative error on the WCST. In the WCST, patients uncover rules so as to form concepts according to the shape, color, and number of cards. The above tasks require logical analysis or visual memory, and the level of gyrus activity may affect the patient's test performance. Furthermore, the ReHo value of the lingual region was positively correlated with the perseverative error of the WCST. The results showed that rTMS may affect the level of ReHo in the lingual gyrus, and thereby affect executive function in patients.

The cuneus is located in the supracalcarinal fissure of the occipital lobe and belongs to the visual cortex. We showed that the level of ReHo in the left and right cuneus of the experimental group decreased, and the level of DC in the right cuneus diminished after rTMS. The ReHo value of the cuneus was positively correlated with time of the WCST, as was the DC value of the cuneus with perseverative error on the WCST and time of the WCST. An increase in the ReHo value in the cuneus was positively correlated with an increase in the anxiety score in the DASS and negatively correlated with an increase in the orientation score (MoCA).The cuneus is principally responsible for processing visual images (40), as both the WCST and Stroop effect color word test require subjects to process visual information, and the cuneus may thus play an important role in the aforementioned tests.

After rTMS, the functional connection strength between the right cuneus and the left cuneus and between the right cuneus and the left and right middle occipital gyrus changed significantly in the experimental group. FC strength between the CUN.R and CUN.L was positively correlated with perseverative error on the WCST, FC strength between the CUN.R and MOG.L was negatively correlated with the RT of the Stroop task, and FC strength between the CUN.R and MOG.R was positively correlated with perseverative error of WCST. The middle occipital gyrus belongs to the visual cortex and is responsible for processing visual images and participating in the brain area associated with verbal declarative memory (40). Therefore, the functional connection strength between the right cuneus and the left cuneus, and the right cuneus and the left and right middle occipital gyrus may play important roles in the above tests. Collectively, these results suggest that rTMS may affect the functional connection strength between the right cuneus and the left cuneus, and the right cuneus and the left and right middle occipital gyrus, and thus affect executive function in patients.

The level of DC in the left postcentral gyrus of the experimental group declined after treatment, the DC value of the postcentral gyrus was positively correlated with perseverative error on the WCST and time of the WCST. After rTMS, the functional connection strength between the left postcentral gyrus and the right inferior temporal gyrus (ITG), the left postcentral gyrus, and the left supramarginal gyrus was significantly different between the two groups. FC strength between the PoCG.L and ITG.R was positively correlated with percent correct responses on the WCST, FC strength between the PoCG.L and ITG.R was negatively correlated with percent non-perseverative errors, and an increase in FC strength between PoCG.L and ITG.R was positively correlated with an increase in the MoCA score. ITG is involved in a variety of advanced cognitive functions, including visual object recognition, decision-making, vocabulary and speech comprehension, decision-making, and emotion regulation (41–44). Some studies have found that in patients with chronic schizophrenia, the reduction in bilateral inferior temporal gyrus volume may lead to semantic multimodal sensory integration and complex visual perception dysfunction (45–47).

The supramarginal gyrus belongs to the inferior parietal lobule and is related to mathematics and logic, and the inferior parietal lobule receives input from the visual and somatosensory cortex. Therefore, the inferior parietal lobule is considered to be an important area for the integration of different modal information. Injury to the inferior parietal lobule affects spatial perception and the integration of vision, movement, and other information (48). Some investigators have found that trauma-related stimuli can induce corresponding memories and often cause strong physical responses that are involved in a variety of somatosensory information (49–51), and these can be remembered in the form of physical sensory information (52).

Studies have shown that abnormal activation of the inferior parietal lobule and postcentral gyrus in patients with PTSD precipitated abnormal sensory information processing in these patients (53, 54). The functional connectivity between the left DLPFC and the left superior parietal lobule, bilateral cuneus, and bilateral supraoccipital gyrus was significantly enhanced in PTSD patients, and there may be abnormal functional connections between the frontal lobe-parietal lobe loop and the frontal-occipital lobe loop in patients with PTSD (55). For example, EEG studies have found that primary sensory function was abnormal in PTSD patients (56–58). Abnormal automatic processing is also related to the high arousal symptoms of PTSD (59, 60), which may trigger upset in PTSD patients when exposed to a complex environment. Although the patients in our study did not meet the diagnostic criteria of PTSD, they all experienced stress-related events prior to admission.

After rTMS, the DC value of the postcentral gyrus decreased in the experimental group, and the functional connectivity strength of the left postcentral gyrus and supramarginal gyrus was negatively correlated with the HAMA score. We suggest that this connection may be related to negative emotional arousal. Our study showed that rTMS may therefore affect the level of DC in the postcentral gyrus and its functional connectivity, and then affect performance on cognitive tests.

### Effects of rTMS on Brain Metabolism in Patients With Stress-Related Depression

Proton magnetic resonance spectroscopy (^1^H-MRS) is a non-invasive method that can measure the levels of important metabolites in specific brain regions, and studies have shown that metabolites in the brain may be related to the pathophysiology of mental illness (61). The primary metabolites that we analyzed with ^1^H-MRS were N-acetylaspartate (NAA), choline (Cho), and creatine (Cr).

We did not uncover any significant difference in the level of Cho in the left DLPFC between the two groups before rTMS, but the level of choline (Cho) in the experimental group was significantly lower than that in the control group after rTMS. Studies have shown that Cho levels and the Cho/Cr ratio in the bilateral DLPFC in young patients with depression were higher than those in healthy controls, especially with respect to the left DLPFC (62), and choline levels in the left DLPFC were abnormally elevated in children and the elderly with severe depression (63, 64). Patients with stress-related depression who participated in the current study may have had high levels of Cho in the left DLPFC, and correlation analysis showed that changes in Cho in the left DLPFC were positively correlated with changes in the RT Stroop task. rTMS may thus improve the executive function of patients by promoting the levels of Cho in the left DLPFC.

Before rTMS, the NAA/Cr ratio in the left DLPFC in the experimental group was significantly higher than that in the control group, and the gap was further augmented after treatment. The patients with a longer duration of depression exhibited a lower NAA/Cr ratio in the left DLPFC relative to those with a shorter course of disease and compared with healthy people (65). The change in memory capability has been positively correlated with the ratio of N-acetylaspartate to creatine (NAA/Cr) and the ratio of choline to creatine (Cho/Cr) in the right brain (66). After high-frequency (15-Hz) rTMS over the left DLPFC in patients with treatment-resistant depression, we noted that an improvement in the number of persistent errors on the WCST after treatment was positively correlated with an increase in NAA levels in the left anterior cingulate gyrus of the rTMS group (38). Our correlation analysis showed that the NAA/Cr ratio in the left DLPFC was positively correlated with a correct-response rate on the WCST. rTMS may therefore improve executive function in patients by stabilizing the NAA/Cr ratio in the left DLPFC.

## Conclusions

Ten-Hz rTMS improved the cognitive function (executive function) of patients with stress-related depression, and this may have been due to two underlying mechanisms. One was a reduction in the activity level of sensation-related brain regions, reducing the functional connection of vision-related brain regions, improving the functional connection between somatosensory cortex and visual-auditory cortex, and stabilizing the functional connection between inferior parietal lobule and pallidus. The other mechanism appeared to be a reduction in the level of Cho and stabilization in the level of NAA/Cr in the left DLPFC.

## Data Availability Statement

The original contributions presented in the study are included in the article/supplementary material, further inquiries can be directed to the corresponding author/s.

## Ethics Statement

The study was performed in accordance with the Declaration of Helsinki and was approved by the Local Ethics Review Committee (Ethics Committee of the Hospital 984 of PLA). The patients/participants provided their written informed consent to participate in this study. Written informed consent was obtained from the individual(s) for the publication of any potentially identifiable images or data included in this article.

## Author Contributions

YuxC designed research, conducted research, performed analysis, and wrote the paper. LQ and YZ designed research, supervised research, reviewed, and revised the manuscript, and granted research funds. XL developed clinical conception. LW performed software and data curation. ST, YuaC, FW, KG, YW, GX, SZ, JL, HW, ZJ, LL, and XiW involved in clinical assessment and rTMS administration. FX, XuW, SW, and CX interpreted data. The authors accept responsibility for the integrity of the data and the accuracy of the data analysis. All authors contributed to the article and approved the submitted version.

## Funding

This work was supported by the National Defense Basic Science Research Program of China (Grant No. JCKY2019548B001) and the National Defense Science and Technology Innovation Project of China (Grant No. 1716312ZT00216401).

## Conflict of Interest

The authors declare that the research was conducted in the absence of any commercial or financial relationships that could be construed as a potential conflict of interest.

## Publisher's Note

All claims expressed in this article are solely those of the authors and do not necessarily represent those of their affiliated organizations, or those of the publisher, the editors and the reviewers. Any product that may be evaluated in this article, or claim that may be made by its manufacturer, is not guaranteed or endorsed by the publisher.
